# First reported human use of wireless laparoscopic system: is it ready for prime time?

**DOI:** 10.1007/s00464-024-11286-9

**Published:** 2024-09-27

**Authors:** Hee Kyung Jenny Kim, Abel Abraham, Jamie DeCicco, AJ Haas, Robert Pollard, Kevin El-Hayek

**Affiliations:** 1 Case Western Reserve University School of Medicine, Cleveland, OH USA; 2 The MetroHealth System, Cleveland, OH USA; 3 Northeast Ohio Medical College, Rootstown, OH USA; 4 Cleveland Clinic Lerner College of Medicine, Cleveland, OH USA

**Keywords:** Wireless laparoscopy, Battery-powered laparoscopy, Surgical technology, Surgical ergonomics, Global surgery

## Abstract

**Objective:**

During the advent of laparoscopy, surgeons directly explored the abdominal cavity with a telescope-like device through a small incision. Since then, numerous technological advances have transformed minimally invasive surgery (MIS). Yet, in our wireless world, various devices crowd the surgical field, with long wires and light sources posing fall and fire risks. The primary objective of this study was to analyze the first reported human use of a novel wireless laparoscopy system or WLS (ArthroFree™, Lazurite®, Cleveland, Ohio).

**Methods:**

The utility and convenience of the WLS was assessed via two avenues: (1) by analyzing surgical outcomes from first human use and (2) by surveying healthcare professionals regarding its quality and utility.

**Results:**

Eighteen patients (mean age 44.2, 83.3% female, mean BMI 33.4) underwent operations with the WLS. Operations included gynecologic and general surgical procedures. There were no intraoperative or postoperative complications, and no conversions to traditional laparoscopy or laparotomy. Mean operating time was 71.94 ± 20.41 min, and estimated blood loss was minimal. Survey results revealed varied individual experiences. Strengths included adequate illumination, improved ergonomics, and simplicity of setup and ease of operation. One respondent criticized the image resolution. Feedback indicated an overall positive impact, and 67% of respondents supported inclusion of the device at their facility. Moreover, its deployment in resource-limited settings abroad has demonstrated its efficacy in global surgery, indicating its potential in various healthcare environments.

**Conclusions:**

This is the first reported human use of a novel WLS. Clinical results supported efficiency and safety of the technology. The successful deployment of the WLS in diverse surgical environments, including resource-limited settings, highlights its potential as a universally adaptable tool in global surgery. This report represents a strong first step toward a wireless operating room with the promise of redefining surgical standards as well as bridging gaps in surgical care worldwide.

**Graphical abstract:**

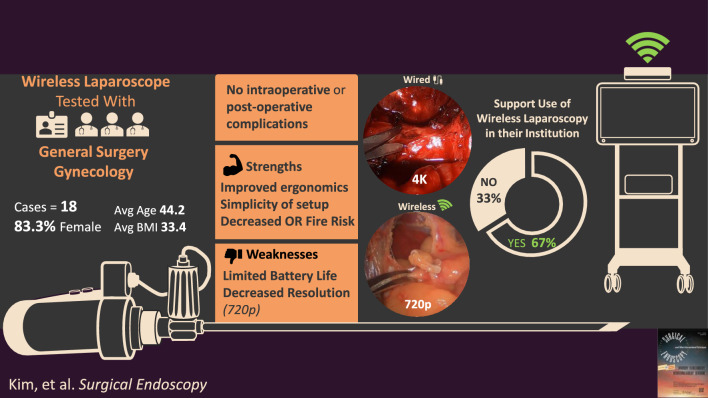

## Background

The invention and subsequent widespread adoption of laparoscopy transformed modern surgery, leading to minimal blood loss, expedited recovery time, and a decreased overall morbidity [1]. In the early days of laparoscopy, a surgeon directly visualized the abdominal cavity with a telescope-like device in a single incision. Since then, numerous advances in technology have transformed laparoscopic surgery [2, 3]. High-definition cameras and fiber optic light cables now enable the surgeon to accurately visualize the surgical field [4, 5]. However, technological advances posed new risks. Today, various equipment and cords can crowd the surgical field, with long wires and light cords posing both fall and fire risks [6, 7]. The sophistication of technology has heightened resource disparities. While laparoscopy is a widespread practice in the United States and other high-income countries, communities without access to reliable electricity are excluded from the benefits of minimally invasive surgery (MIS).

This WLS includes a wireless laparoscopic camera and a high-intensity light source. The system is battery-powered, eliminating at least two power cords from the operating room. The wireless camera head, along with the light source, can be attached to any regular laparoscope. The image transmits wirelessly to the remote receiver unit which can connect to a surgical monitor screen. This system is the first technology of its kind with Federal Drug Administration approval. The technology aims to address limitations posed by traditional laparoscopic cameras, such as poor heat dissipation, cumbersome cords, and reduced surgical ergonomics. In this study, we describe the first uses of wireless laparoscopy in a single institution, assess the utility and convenience of the technology, and survey healthcare workers’ perception.

## Materials and methods

A retrospective review of patients was conducted of patients who underwent wireless laparoscopy from August 2023 to January 2024. Patient demographics and operative data were studied. Surgeon and healthcare workers’ perception of the new technology was assessed utilizing a standardized and anonymized survey. This study was approved by the MetroHealth System Institutional Review Board.

**Fig. 1:**
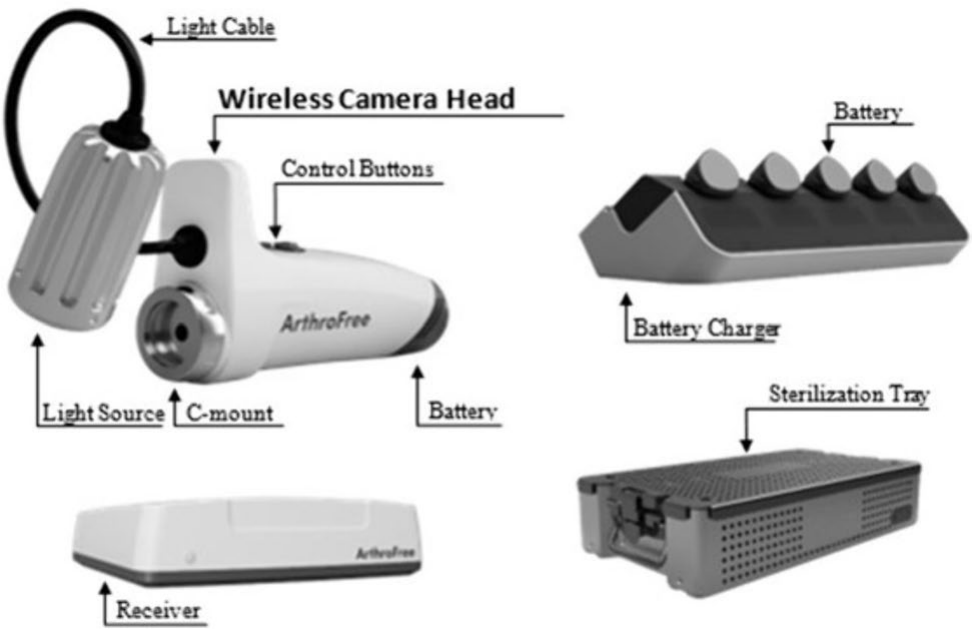
ArthroFree™ wireless laparoscopy components

The ArthroFree™ WLS (Fig. 1) consists of a high-definition camera head and remote receiver unit. The receiver unit has two digital outputs. Images are transmitted to the remote receiver unit which is compatible with most existing monitors. Standard laparoscope lenses can be used with this laparoscope. The camera features include white balance, adjustable light intensity, image capture and recording, and zoom. The camera head has an advanced light source that minimizes heat generation. The maximum temperature of ArthroFree’s light source is 43 °C. The camera head has a battery that is external, removable, and rechargeable. The battery life is one hour, with at least 300 charges (Table 1).

**Table 1:** Patient characteristics

Characteristic	Value
Total	18
Age (mean ± SE)	44.2 ± 4.35
Female (*N*, [%])	15 [83.3%]
Race (*N*, [%])	
White non-hispanic	9 [50.0]
Black or African American	7 [38.9]
Declined to answer	2 [11.1]
BMI (mean ± SE) kg/m^2^	33.4 ± 6.48

## Results

**Fig. 2:**
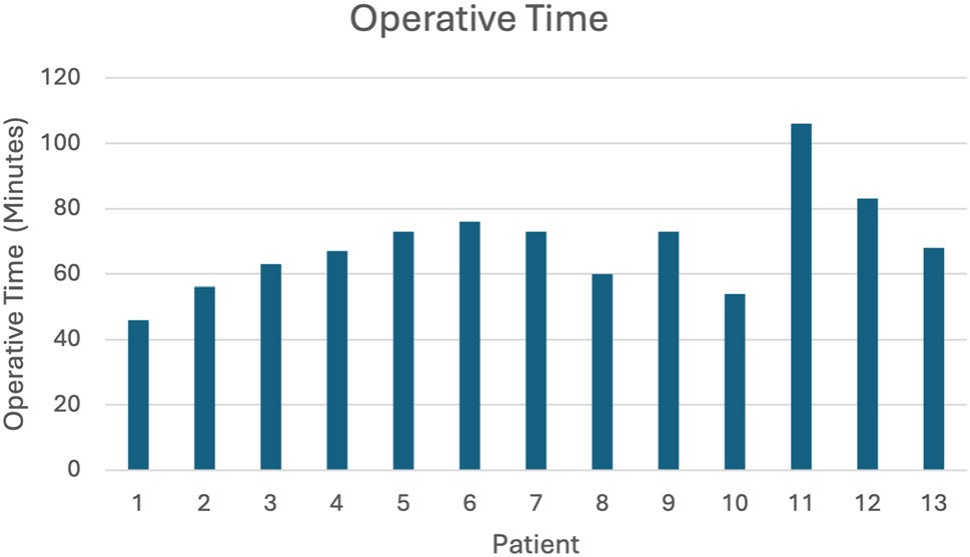
Operative time of each procedure

**Fig. 3:**
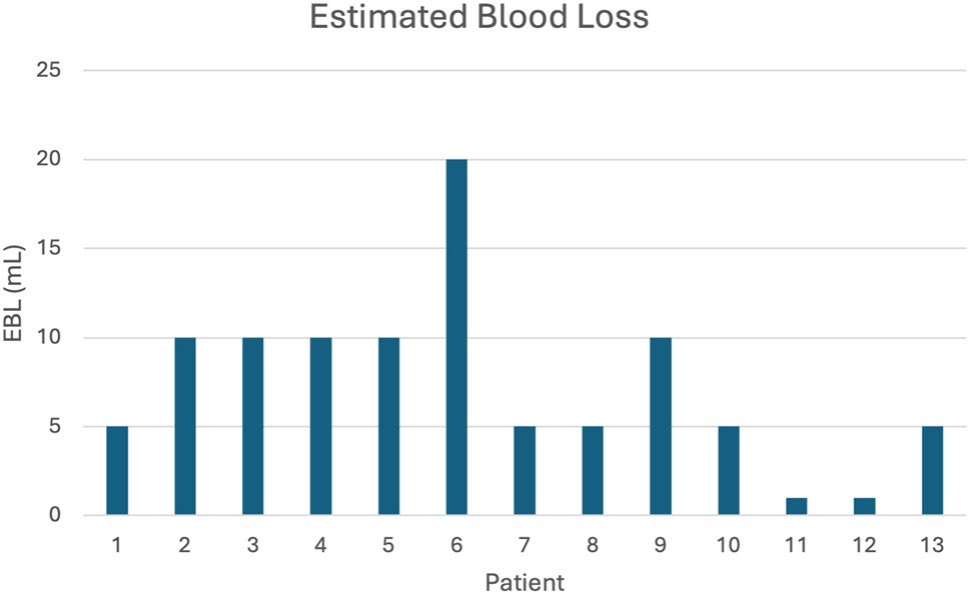
Estimated blood loss of each procedure

The utility and convenience of the WLS system were assessed in two avenues: (1) by studying the operative course and (2) by surveying healthcare professionals. Eighteen patients (mean age 44.2 years, 83.3% female) underwent operations with this WLS. The mean BMI was 33.4 kg/m^2^. Patients were 50% White/Non-Hispanic, 38.9% Black or African American, and 11.1% of the patients declined to answer. The operations included various gynecologic and general surgery procedures (Table 2). Mean operating time was 71.94 (± 20.41) minutes, and estimated blood loss was 11.78 (± 11.89) mL (Figs. 2, 3). No intraoperative or postoperative complications were noted, and no cases were converted to traditional laparoscopy or laparotomy.

**Table 2:** Procedures performed with wireless laparoscopy

Operation type	Number of cases
Cholecystectomy	11
Hernia repair	2
Endometriosis excision	2
Diagnostic laparoscopy	1
Hysterectomy	4
Salpingo-oophorectomy	4
Cystoscopy	1
Cystourethroscopy	2
Colpopexy	1
Oophoropexy	1

Survey respondents included two attending surgeons and one surgical staff member. The surgery staff member noted that the wireless laparoscope led to an easier setup, use, and takedown, deeming it easier to work with compared to the traditional laparoscopic camera. One surgeon found that the WLS reduced their stress during the procedure and made them more efficient. On evaluation of camera performance, one surgeon found that the display quality was not appropriate for the procedure, making the WLS clinically inequivalent to their current laparoscopic camera. All survey participants indicated that they were satisfied with other technical aspects of the camera such as its weight, ergonomic comfort, and design of the buttons. The wireless light was adequate for the procedures according to all respondents. Overall, individual experienced varied; 67% agreed or strongly agreed to adding the device to their facility while 33% answered neutral. All respondents, however, agreed that it is important to minimize the number of items in the sterile field.

## Discussion

This the first reported human use of a novel wireless laparoscopy system. The ArthroFree™ System was utilized in a variety of abdominal and gynecologic procedures. The reduction in clutter was desirable. Having fewer wires also allowed surgeons to optimize ergonomics during the procedures. Wires also contribute to clutter within the operating room, subsequently posing as fall hazards, potentially compromising provider and patient safety [7]. In addition, the heat emitted from the optical light cord may cause operating room fires, as its temperature has been shown to reach up to 268 °C [6].

Beyond the safety benefits, eliminating the light and power cords leads to a greater range of motion and overall satisfaction by users [8]. Our findings were similar in that most respondents reported increased comfort and convenience associated with the WLS.

**Fig. 4:**
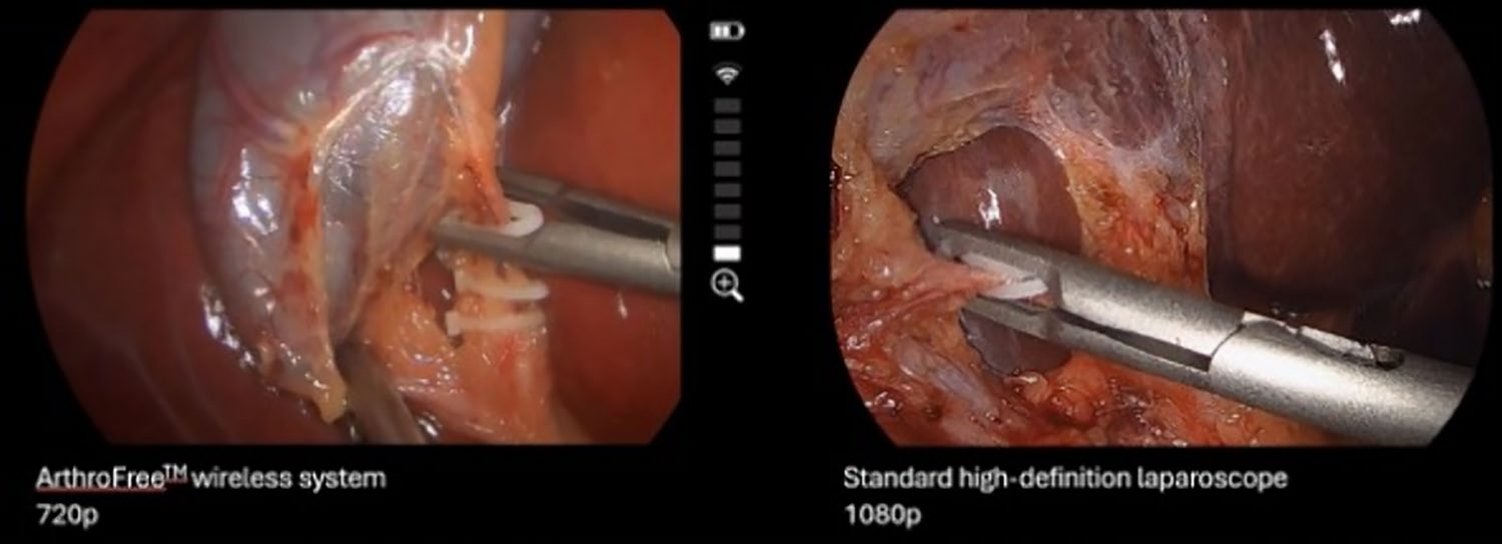
Comparison of the 720p resolution of the WLS and the 1080p resolution of the standard laparoscope

Though all those surveyed agreed that it is important to have the fewest items possible in the sterile field, this WLS fell short of some expectations, namely the video quality. The camera resolution is at 720p while the resolution of typical laparoscopic cameras is 1080p or 4 K (Fig. 4). Video quality is an important piece of the surgeon experience; the difference between 720 and 1080p however did not seem to affect short-term patient outcomes in preliminary results of a study by Zhang et al. [9] Nonetheless, ensuring that the transmitted images are of higher definition must be one of the key target areas of improvement for the WLS. Like the findings from Zhang et al. our patient outcomes were notable for no intraoperative or postoperative complications, supporting the safety of the technology. No conversion to laparotomy or traditional laparoscopy supports the efficacy of the technology. We do not anticipate any technical challenges in the adaptation of the new technology when used on more basic laparoscopic cases. More complex operations may require higher definition devices given the nuances in anatomy that one can see with higher quality video. That said, surgeons have successfully incorporated other wireless technology, such as wireless ultrasonic devices, into current practice [10].

Our findings are inherently valuable as they represent the first evaluation of wireless laparoscopy technology in humans. We assessed its use in a variety of surgical cases, both general surgery and gynecology. Limitations of the study include the small sample size and few responses to the survey. As the technology evolves, expanding our study to have a larger sample size will be a natural next step.

This study demonstrated numerous benefits to wireless laparoscopy. The potential benefits of wireless laparoscopy extend beyond its use in our institution, an academic medical center in an urban area of a high-income country (HIC). In a study by Chawla et al. that reviewed 21 low-to-middle-income countries (LMICs), less than two-third of hospitals had a continuous, reliable, power source, or access to an electric generator [11]. All hospitals included in the study provided surgical services.

Equitable access to laparoscopic surgeries in LMICs is impossible without addressing barriers unique to these communities. The battery-powered laparoscope is an apt solution for challenges that stem from unreliable energy. Batteries can be bought or re-charged during times where power is available. The wireless laparoscope can function through power outages. Battery-powered surgical and medical technologies, such as a CT scanner, have proven useful in global health, global surgery, and mass casualty settings [12].

The prohibitive costs of laparoscopic surgical equipment can be mitigated by a portable system such as the reported WLS. One set of battery-powered laparoscopic camera and light source could be utilized in many sites. Lukish and Ellis-Davy describe the successful use of a battery-powered light along with universal serial bus (USB)-enabled laparoscopic camera in remote areas in LMICs during a pediatric general surgical mission [13]. The reported WLS also has been deployed in similar surgical missions abroad with similar success. The primary surgeon traveled with the system and used them during a recent mission to Nigeria without difficulty.

## Conclusions

This is the first reported human use of a novel wireless laparoscopic system. The innovation and versatility has potential to be a game-changer in MIS. Clinical results supported efficiency and safety of the technology, as operative times were similar to traditional laparoscopy, no complications were observed, and there were no challenges unique to the system that affected patient care. While currently optimized for ergonomic efficiency, future enhancements will focus on elevating its display resolution to rival or surpass existing high-resolution laparoscopes. The successful deployment of ArthroFree™ in diverse surgical environments, including resource-limited settings, highlights its potential as a universally adaptable tool in global surgery. This study represents a strong first step toward a wireless operating room with the promise of redefining surgical standards as well as bridging gaps in surgical care worldwide.
